# A systematic audit of transparency and validation disclosure in commercial veterinary artificial intelligence

**DOI:** 10.3389/fvets.2026.1761038

**Published:** 2026-03-05

**Authors:** David Brundage

**Affiliations:** School of Veterinary Medicine, University of Wisconsin-Madison, Madison, WI, United States

**Keywords:** clinical decision support systems, diagnostic imaging AI, generative AI (GenAI), good machine learning practice, veterinary artificial intelligence

## Abstract

**Objective:**

To systematically identify the commercial market for clinical artificial intelligence (AI) products in veterinary medicine and audit their public documentation for transparency using a standardized, evidence-based instrument.

**Methods:**

A cross-sectional systematic audit of commercial AI tools was completed via a multi-channel search. Inclusion criteria required commercially available products with explicit AI claims and clinical functionality; administrative and direct-to-consumer tools were excluded. Publicly available documentation was archived and evaluated using a 25-point framework adapted from FDA and GMLP guidelines to assess data provenance, validation, safety, and usability.

**Results:**

Seventy-one AI products, available in the North American market were included, comprising Generative and Ambient (*n* = 47), Diagnostic Imaging (*n* = 19), and Specialized tools (*n* = 5). The mean unweighted transparency score across the cohort was 6.4%. Notably, 63.3% (*n* = 45) of vendors failed to disclose a single metric. Diagnostic Imaging tools achieved a higher mean risk-weighted transparency score (13.1%) compared to Generative and Ambient tools (1.8%). While 36.8% of imaging vendors provided peer-reviewed or internal validation evidence, only 2.1% of generative vendors did so. Only one vendor (1.4%) disclosed training data signalment (species, breed, age, sex) or subgroup performance.

**Conclusions:**

The commercial veterinary AI market operates with systemic opacity. This audit reveals a significant “Transparency Gap”—a divergence where the sophisticated clinical capabilities marketed to veterinarians far exceed the publicly available evidence required to validate them. A significant gap exists between maturing imaging applications and unvalidated generative tools. The universal failure to report training demographics renders independent assessment of algorithmic bias impossible.

**Clinical relevance:**

Veterinarians currently bear the legal and ethical burden of validating AI tools without access to necessary performance data. The implementation of standardized transparency frameworks is urgently required to support evidence-based product selection and prevent patient harm from unvalidated technologies.

## 1 Introduction

### 1.1 The emergence of artificial intelligence in veterinary clinical practice

Artificial intelligence (AI) has emerged as a transformative force in modern veterinary medicine, introducing a new class of diagnostic and clinical support tools poised to fundamentally alter professional practice (1, 2). The rapid proliferation of these technologies presents both unprecedented opportunities to enhance animal care and significant new challenges for the profession (1). As AI systems become more integrated into clinical workflows, it is imperative for the veterinary community to critically evaluate their implementation, efficacy, and the frameworks—or lack thereof—governing their use (3).

The key areas of AI application are diverse and continue to expand, promising substantial benefits to clinicians, patients, and clients (1, 3–5). Diagnostic Imaging has seen the most rapid growth, specifically in veterinary radiomics, where AI algorithms are trained to analyze radiographs, computed tomography (CT), and magnetic resonance imaging (MRI) (1, 3, 4, 6, 7). These tools are designed to improve disease detection, assist in characterization, and automate measurements, offering a new layer of support for both general practitioners and specialists (1, 4, 7). Beyond imaging, Clinical and Diagnostic Support systems are increasingly applied to aid in diagnosis, disease surveillance, and epidemiology (1, 4, 5, 8). Algorithms can analyze vast datasets to identify patterns, predict disease outbreaks, and assist in developing treatment strategies, including the planning of complex surgical procedures (1–3, 6). Ultimately, the integration of these tools aims to improve Patient Outcomes by enhancing clinical efficiency and maximizing diagnostic accuracy. By augmenting professional capabilities and streamlining workflows, these technologies aim to elevate the standard of care and advance animal health and welfare (1, 3, 6).

### 1.2 The regulatory vacuum

A significant disparity exists in the regulatory oversight for AI between human and veterinary medicine (9, 10). In human healthcare, established regulatory frameworks and guidelines have been developed to ensure the safety and efficacy of new technologies (9, 11–14). Precedents such as the “Good Machine Learning Practice for Medical Device Development” principles and robust reporting standards for clinical trials illustrate a mature approach to oversight that prioritizes patient safety and evidence-based implementation (9, 15).

In stark contrast, the regulatory landscape for veterinary AI is largely undefined (1, 2, 9). In North America, there are currently no federal pre-market approval requirements for AI tools used in veterinary medicine, which would often be classified as Software as a Medical Device (SaMD) in human medicine (7, 10, 12). This absence of pre-market review does not mean these tools are unregulated; rather, it shifts the regulatory focus from the device to the user. While the AI device itself may not require federal approval, the licensed veterinarian's use of that device is governed by existing standards of professional practice (2, 3, 16). This critical distinction places veterinarians in a professionally precarious position, as they are accountable for using tools that have no mandated, independent pre-market validation.

This lack of a mandatory pre-market review process creates a direct and critical consequence: companies bringing veterinary AI products to market are under no federal obligation to report or publicly disclose validity and performance data (10). This stands in direct opposition to the human medical field, where such transparency is a cornerstone of regulatory approval and clinical trust.

### 1.3 The imperative for transparency and trust

In the context of this regulatory vacuum, transparency becomes critically important. Many advanced AI models function as a “black box,” where the internal decision-making processes are opaque even to their developers, let alone the end-user. For a clinician to ethically and effectively use an AI-generated recommendation, they must be able to understand the reasoning behind it (10, 17, 18). This understanding is essential for establishing trust, verifying clinical validity, and assuming professional responsibility for patient care. However, without access to robust validation data, veterinarians are forced to rely on “experiential opinion” to assess tool performance. This reliance creates a dangerous cognitive trap: in the absence of contradictory evidence, clinicians are highly susceptible to automation bias, a phenomenon where users uncritically accept the outputs of an automated system as objective truth because they lack the necessary metrics to challenge them (19). Consequently, a lack of transparency creates not only a barrier to clinical adoption but a direct mechanism for patient harm. Without insight into how an algorithm functions or performs, veterinarians cannot assess its reliability or identify potential weaknesses, such as algorithmic bias or poor performance on specific patient populations. This fundamental conflict—between the rapid commercialization of opaque technologies and the professional imperative for evidence-based practice—demands a systematic investigation into the actual state of transparency in the veterinary AI market.

### 1.4 A lack of standardized transparency in commercial products

Despite the rapid commercialization of AI tools for veterinary clinical practice (20), the level of transparency provided by their vendors represents a critical vulnerability for the profession (2, 21). We characterize this as the “Transparency Gap”: the widening disparity between the advanced clinical capabilities promised in marketing materials (e.g., automated diagnosis, treatment recommendations) and the lack of publicly available documentation required to clinically validate those claims. The accelerating adoption of these technologies has created a chasm between technological capability and evidence-based practice, confronting veterinarians with a fundamental information deficit that complicates decision-making and places an undue burden of validation on the individual practitioner (3, 10, 21).

While numerous commercial products are now on the market, there has been no systematic audit or application of a standardized metric to assess the level of transparency provided in their public documentation (2, 10, 16, 22). This gap is starkly articulated by previous analyses of the commercial landscape, for example, Joslyn and Alexander report that “no clinically available veterinary radiology AI algorithms have peer-reviewed evidence of clinical efficacy or publicly listed product specifications and performance measures” to allow for their independent evaluation (2, 7).

### 1.5 Study aim and approach

The burden of due diligence thus falls entirely on the individual practitioner, who often lacks the specific expertise in data science required for a rigorous assessment of algorithmic tools (2, 3, 7, 10, 21) This profound information asymmetry is a direct consequence of an industry-wide failure to provide standardized, public-facing evidence of algorithmic performance, training, and validation (2, 3, 7, 10, 21). As a result, veterinarians are left unable to make fully informed, evidence-based purchasing and clinical decisions (3, 10). To address this critical knowledge gap, this study conducts, to our knowledge, the first systematic audit of transparency in the commercial veterinary AI landscape.

This study is designed as a direct response to the gap between the rapid commercial proliferation of veterinary AI and the lack of standardized transparency from its vendors (10, 21). The primary aim is to systematically identify commercially available clinical AI products in veterinary medicine and audit their public documentation for transparency using a standardized, evidence-based instrument. This audit was limited to publicly accessible marketing and documentation materials; we acknowledge that vendors may provide additional technical information through sales or contractual channels that are not captured by this methodology.

The scope of this investigation is focused strictly on commercially available clinical AI applications intended for use by veterinary professionals in a clinical setting and currently marketed to practitioners in North America. This includes tools for diagnostic imaging, clinical decision support, and all other applications directly involved in patient diagnosis, management, and treatment. Administrative tools (e.g., billing or scheduling software) and direct-to-consumer products are excluded from this analysis, as they do not directly inform or augment the clinical decision-making process under the purview of a licensed professional. By quantifying the level of transparency across the commercial landscape, this study will provide a crucial baseline for the veterinary profession. Its contribution is intended as a foundational step toward establishing benchmarks for transparency and accountability, empowering veterinarians to make more informed decisions and encouraging a market that prioritizes evidence and trust.

## 2 Materials and methods

### 2.1 Search strategy

A systematic, multi-channel search strategy was employed to identify the commercial landscape of veterinary AI products utilizing the Preferred Reporting Items for Systematic Reviews and Meta-Analyses (PRISMA) as a guiding framework to structure the search and selection process (23). Data collection was performed during a discrete 7-day window from November 17, 2025, to November 24, 2025. While many of these vendors distribute software globally, the search strategy utilized US-based search engines and focused on North American veterinary conference exhibitor lists, effectively centering the audit on the North American markets. This condensed timeframe was selected to establish a strict cross-sectional snapshot, minimizing the temporal confounding that arises from rapid software versioning and documentation updates during the data extraction period.

To minimize selection bias and capture the full spectrum of the market—from established industry leaders to early-stage startups—four distinct data sources were queried.

1. Conference exhibitor lists: public vendor lists from three major veterinary conferences (VMX 2025, WVC 2025, and AVMA Convention 2025) were screened to identify companies with active commercial operations.
2. Business aggregators: Crunchbase and LinkedIn were searched to identify venture-backed startups not yet exhibiting at major conferences. Queries included “veterinary healthcare” filtered by keywords “Artificial Intelligence,” “Machine Learning,” and “AI.”
3. Mobile app stores: the Apple App Store and Google Play Store were searched using the terms “Veterinary Artificial Intelligence” and “Veterinary AI” to capture point-of-care mobile tools.
4. General web search: a structured Google search was performed using an exclusionary string (veterinary OR “animal health”) AND (“artificial intelligence” OR “machine learning”) -site:.edu -site:.gov -“journal of” to filter out academic literature and isolate commercial entities.

The first 100 results per query were screened. Results were consolidated into a master database, and duplicates were removed using a hierarchical matching process based on product name and URL.

### 2.2 Eligibility criteria

To keep a clear boundary between clinical AI and administrative AI, strict inclusion and exclusion criteria were developed. To be included, a product was required to: (1) be commercially available (defined as having an active website with a mechanism for purchase or demo scheduling); (2) explicitly claim the use of AI or machine learning; and (3) perform a clinical task, such as diagnosis, triage, image segmentation, or risk prediction. Generative AI/Ambient Scribes were included only if they performed clinical summarization, restructured medical records (SOAP notes), or generated differential diagnoses, thereby acting as Clinical Decision Support (CDS) tools as defined by Assistant Secretary for Technology Policy (24).

Products were excluded if they were: (1) purely administrative (e.g., appointment scheduling, inventory management); (2) Direct-to-Consumer (DTC) apps targeting pet owners rather than veterinary professionals; or (3) Academic prototypes or “coming soon” pages with no route to access. All data modalities and clinical domains were included.

### 2.3 Forensic web archiving and data extraction

To ensure reproducibility and mitigate the risk of “stealth editing” or content removal during the review period, a forensic digital preservation protocol was implemented. On November 28, 2025, an automated pipeline captured a time-stamped, offline mirror (WARC file) of every included vendor's website (see Supplementary Appendix S1 for archiving specifications). Data extraction was performed exclusively on these static snapshots to ensure a consistent cross-sectional analysis. If a data element was not found in the archived documentation, it was recorded as “not publicly available,” consistent with regulatory audit standards where the absence of evidence is treated as evidence of absence.

### 2.4 The transparency audit instrument

To systematically assess the captured data, we developed the Veterinary AI Transparency Index (VATI). This 25-point scorecard was adapted from established regulatory frameworks in human healthcare, including the FDA's Good Machine Learning Practice (GMLP) guiding principles, CONSORT-AI, and the CHAI Model Card framework (11, 25, 26). To ensure content validity, each of the 25 audit items were mapped directly to a corresponding requirement in these guidelines.

The VATI evaluates vendors across four distinct domains: (1) Data Provenance and Composition, which characterizes the model's inputs, including training volume and breed/species representation; (2) Performance and Validation, which assesses the rigor of testing, specifically the use of independent test sets and the reliability of outputs; (3) Safety and Risk Management, which captures the disclosure of known risks, limitations, bias evaluations, and failure modes; and (4) Usability and Documentation, which evaluates the availability and clarity of user manuals and intended use statements (Table 1).

**Table 1:** The veterinary AI transparency index (VATI) framework adapted from human regulatory standards.

**Audit metric**	**Human standard source**	**Justification and clinical relevance**
**Data provenance and composition**		
Training data volume	GMLP 3—Representative patients/data	Small datasets → model memorizes training set, fails on new cases.
Data provenance (source)	CONSORT-AI 5(ii)—Input data	Case mix (referral vs. general practice) may not match your clinic.
Patient demographics	GMLP 3—Representative patients/data	Breed/species mix and phenotypic diversity may not match your patients.
Geographic/site diversity	GMLP 3—Representative patients/data	Regional disease prevalence differences affect false positives/negatives.
Data temporality (dates)	CONSORT-AI 6a—Recruitment dates	Older data (e.g., 2010 vs. 2024) may not reflect current practice.
Reference standard	GMLP 1—Multidisciplinary oversight	Training labels from specialists vs. non-experts determine label reliability.
Data exclusion criteria	CONSORT-AI 4a(ii)—Input-level criteria	Excluding poor-quality/difficult cases reduces robustness in real-world use.
**Performance and validation**		
Performance metrics	GMLP 7—Human–AI team performance	Concrete metrics (Se/Sp/AUC) needed, not vague “high accuracy” claims.
Confidence intervals	CONSORT-AI 17a—Precision/CI reporting	Narrow vs. wide confidence intervals change how “90% accuracy” is interpreted.
Independence of test set	GMLP 4—Independent train/test sets	Train–test overlap (same animals) artificially inflates performance.
External validity	GMLP 8—Clinically relevant testing	Tested on truly external data (new clinics/devices) shows real-world fit.
Subgroup analysis	GMLP 8—Clinically relevant testing	Performance must be checked across species, breeds, ages, and other subgroups.
Human benchmark	GMLP 7—Human–AI team performance	Performance benchmarked vs. generalists and specialists calibrates trust.
Peer-reviewed validation	Academic standard	Peer-reviewed evidence supports claims in an otherwise unregulated market.
**Safety and risk management**		
Limitations/failure modes	GMLP 9—Clear user information	Clear “do not use” conditions prevent misuse (e.g., foals, bandages).
Bias evaluation	GMLP 8—Clinically relevant testing	Evaluation for systematic underperformance on certain phenotypes.
Out-of-distribution handling	GMLP 8—Clinically relevant testing	System can abstain or reject nonsense/unknown inputs instead of forcing a diagnosis.
Risk mitigation/guardrails	GMLP 2—Software/security practices	Human-in-the-loop, edit, and override options protect against automation errors.
Post-market feedback	GMLP 10—Post-market monitoring	Easy mechanisms to report errors/near misses enable post-deployment monitoring.
**Usability and documentation**		
Model design (architecture)	CONSORT-AI 5a—Algorithm type	Declared model type (e.g., CNN, LLM) and input modality.
Explainability	GMLP 7—Human–AI team performance	Explanations (heatmaps, citations) let users verify reasoning.
Output confidence	GMLP 7—Human–AI team performance	Calibrated confidence or risk scores to weigh AI recommendations.
Model card availability	GMLP 9—Clear user information	Standard “AI label” format to compare vendors consistently.
Regulatory clearance	FDA 510(k) status	Indicates FDA clearance/authorization vs. enforcement discretion or no review.
User instructions	GMLP 9—Clear user information	Clear acquisition and use instructions to achieve validated performance.

To simulate the real-world “pre-purchase” due diligence performed by a veterinary clinician or hospital administrator, this audit was strictly limited to public-facing documentation available without barriers to entry. “Publicly available” was defined as information accessible without the requirement to: (1) purchase the software; (2) schedule a sales demonstration; (3) sign a Non-Disclosure Agreement (NDA); or (4) contact the vendor for a white paper.

To ensure methodological consistency and mitigate the risk of interpretative bias, the VATI instrument was designed to prioritize “low inference” metrics governed by strict adjudication criteria. A standardized scoring rubric (Supplementary Appendix S2) was employed to distinguish between verifiable evidence and marketing assertions. Transparency credits were awarded only for specific, quantitative, or verifiable disclosures. Qualitative marketing claims—such as “world-class accuracy,” “unmatched precision,” “veterinarian-approved,” or “tested on thousands of cases”—were explicitly excluded and scored as non-disclosure (0). Conversely, a metric was scored as disclosed (1) only if it met a strict evidentiary threshold, such as the provision of discrete numerical values (e.g., “Sensitivity: 92%,” “Training Set: 10,000 images”), the reporting of statistical uncertainty (e.g., confidence intervals), or the citation of bibliographic evidence detailing the validation study. Isolated statistical claims appearing in marketing materials were scored as non-disclosure (0) unless accompanied by supporting evidentiary context, such as confidence intervals, sample sizes, or a citation to a validation study.

This binary approach minimizes grader subjectivity by requiring objectively verifiable data points rather than qualitative assessments of study quality. To quantify the reproducibility of this extraction protocol, a random stratified sample of 20% of the included vendors (*n* = 14) was re-audited by the primary reviewer following a 7-day washout period. This re-evaluation was performed blindly to the initial scores. Intra-rater reliability was calculated using Cohen's Kappa coefficient (κ), yielding high internal consistency (κ>0.90) and confirming the stability of the extraction criteria.

### 2.5 Statistical analysis

Two scoring indices were calculated for each vendor: an Unweighted VATI (raw percentage of disclosed items) and a Risk-Weighted VATI, which prioritized safety-critical features. In the weighted model, “Safety” metrics (e.g., bias testing, failure modes) received 3 points, “Validation” metrics received 2 points, and “Administrative” metrics received 1 point. The weighting scheme was derived from the risk categorization principles established by the International Medical Device Regulators Forum (IMDRF) for Software as a Medical Device (SaMD). Consistent with the IMDRF's position that “testing of software is not sufficient to determine that it is safe in operation,” we assigned the highest weight (3 points) to Safety and Risk Management metrics (e.g., bias evaluation, failure modes), as these represent the “built-in confidence” measures required to prevent patient harm. Performance and Validation metrics (e.g., sensitivity, specificity) were weighted second (2 points) as necessary but insufficient evidence of reliability. Administrative metrics (e.g., model definition, versioning) were weighted lowest (1 point) as they represent descriptive declarations rather than empirical evidence of safety (27, 28).

Vendors were stratified into three clinical domains: Diagnostic Imaging AI (Pixel-based), Generative and Ambient AI (NLP/Text-based), and Specialized/Other. Differences in transparency scores between domains were assessed using the Kruskal–Wallis *H*-test with *post-hoc* Mann–Whitney *U*-tests (Bonferroni-corrected). Categorical differences in the reporting of specific high-priority metrics were evaluated using Fisher's Exact Test. All analyses were performed using Python (v3.12; Pandas/SciPy). Specialized/Other vendors were excluded from pairwise statistical comparison due to insufficient sample size.

## 3 Results

### 3.1 Search results and market composition

**Figure 1:**
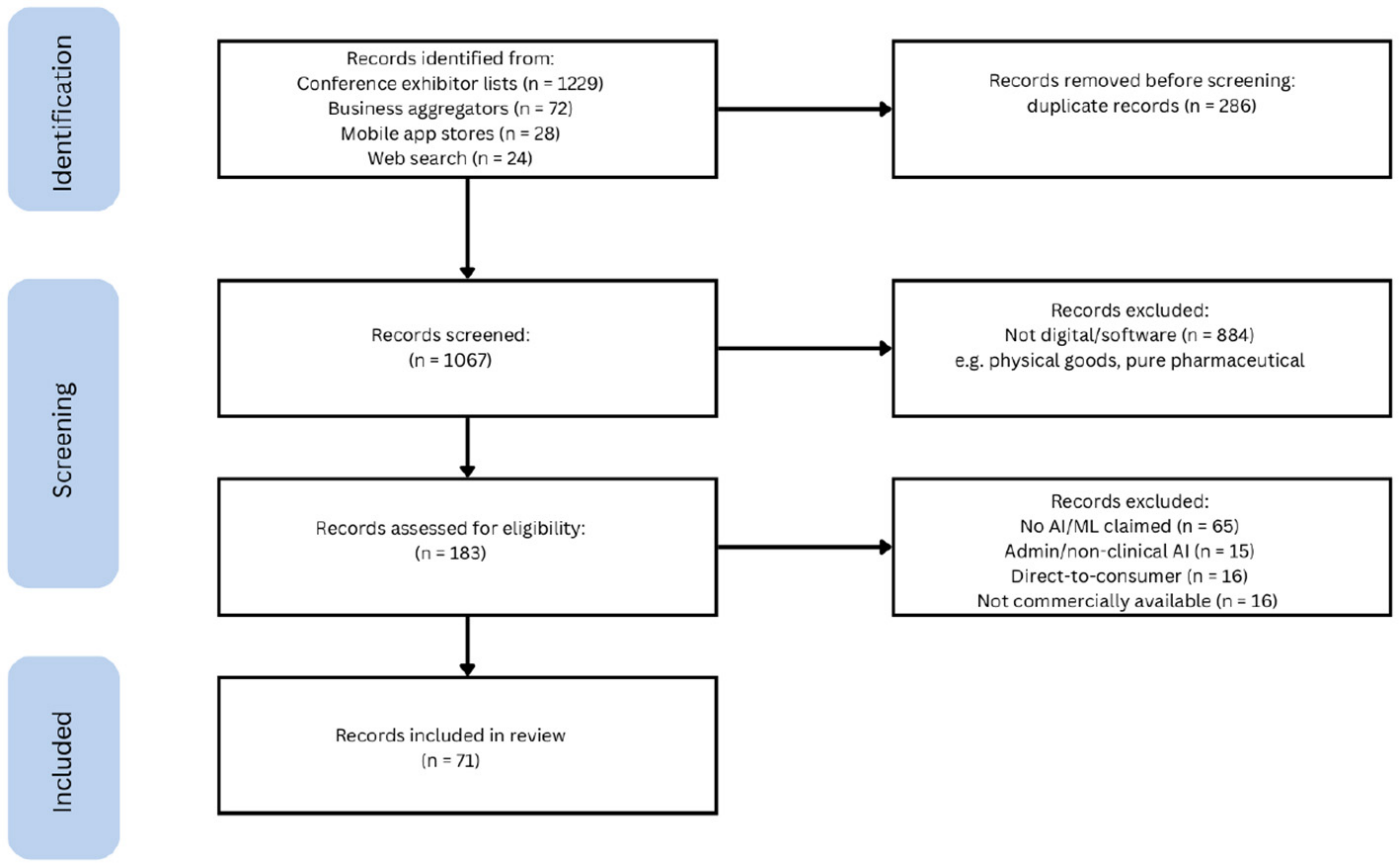
PRISMA flow diagram summarizing the search and selection process.

The systematic search strategy identified 1,353 initial records. Following the removal of duplicates and non-digital products, 183 records were assessed for eligibility. Of these, 112 were excluded for failing to meet inclusion criteria, primarily due to a lack of explicit AI/ML claims or non-clinical functionality (Figure 1).

To facilitate analysis, vendors were stratified into three distinct functional domains based on their primary input modality and clinical utility. Diagnostic Imaging AI was defined as pixel-based computer vision algorithms designed to analyze radiographs, computed tomography (CT), or magnetic resonance imaging (MRI) for the purposes of lesion detection, segmentation, and classification. Generative and Ambient AI encompassed natural language processing (NLP) tools and Large Language Models (LLMs) designed to process or generate text, including ambient clinical scribes, automated record summarizers, and chatbots for differential diagnosis support. Finally, Specialized AI captured domain-specific tools targeting niche diagnostic workflows outside of general radiology or documentation, specifically applications for precision medicine, and precision oncology.

**Figure 2:**
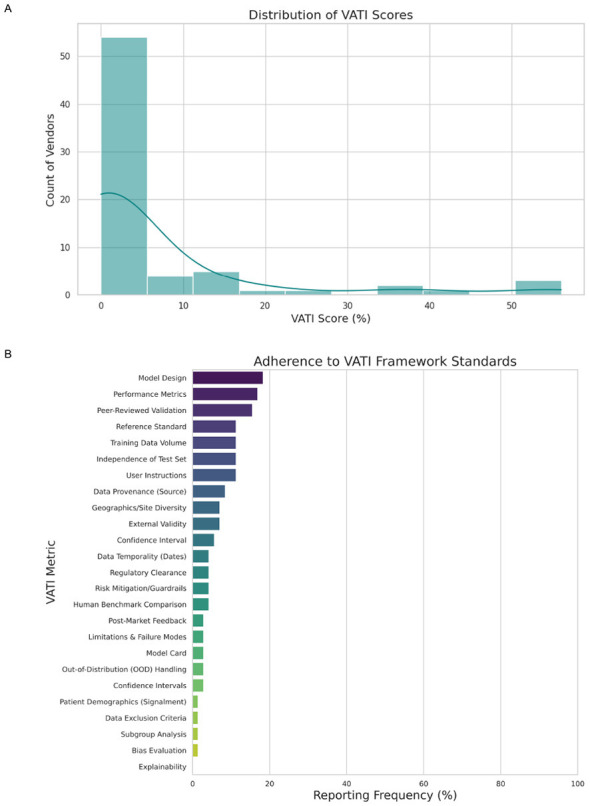
**(A)** Distribution of veterinary artificial intelligence transparency index (VATI) scores and adherence to VATI framework standards. **(B)** Reporting frequency per metric for all vendors.

A total of 71 commercially available AI tools were included in the final audit. The market composition was dominated by Generative and Ambient AI tools (66.2%, *n* = 47), followed by Diagnostic Imaging AI (26.8%, *n* = 19) and Specialized applications (7.0%, *n* = 5). Overall, transparency performance across the cohort was negligible. The mean unweighted transparency score was just 6.4% (range: 0.0%–55.6%), with a heavily zero-inflated distribution. Notably, 63.3% (*n* = 45) of vendors failed to disclose a single metric from the audit framework; for these products, no public information regarding model development, validation, or safety was available to the consumer (Figure 2).

### 3.2 Veterinary artificial intelligence transparency

**Figure 3:**
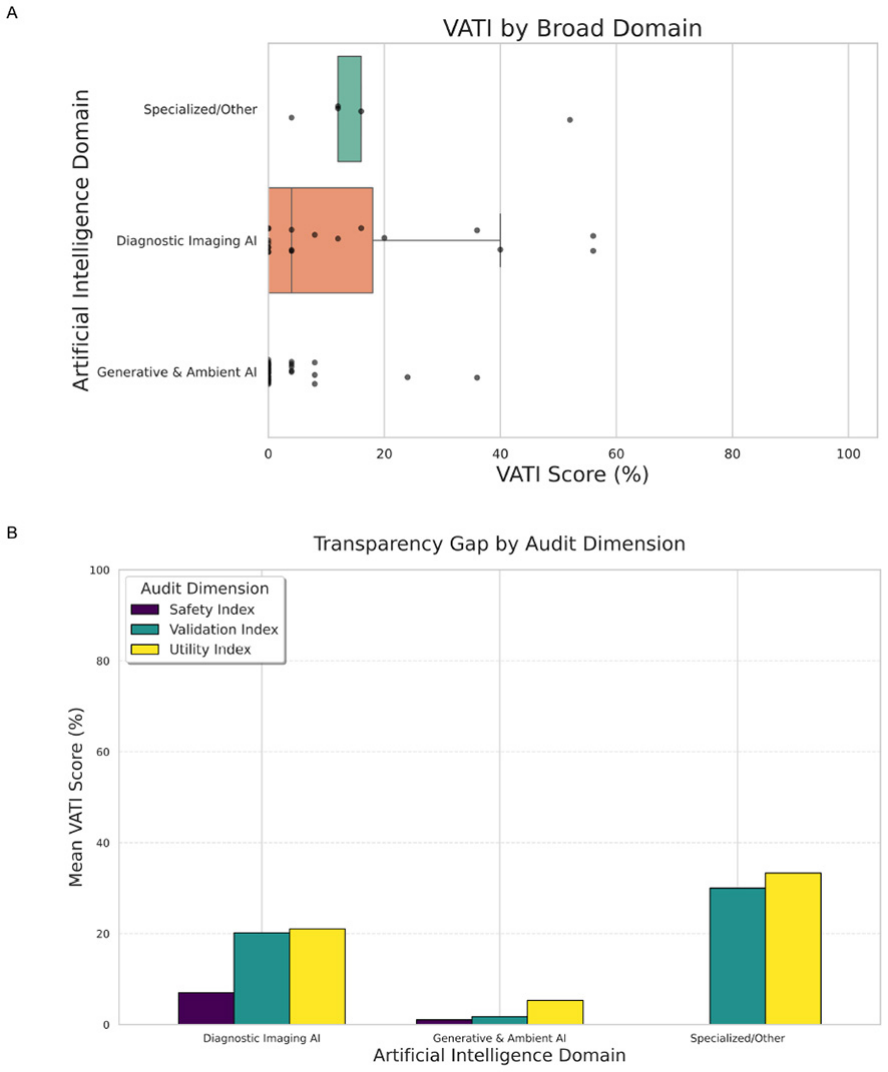
**(A)** Veterinary artificial intelligence transparency index (VATI) scores by broad domain. **(B)** Transparency gap by audit dimension.

When transparency scores were weighted according to clinical risk (VATI-Weighted), a significant and polarizing disparity emerged between modalities (Kruskal-Wallis, *p* < 0.001). Diagnostic Imaging AI tools achieved a mean weighted score of 13.1%. While still low, this represented significantly higher reporting rates compared to the sector of Generative and Ambient AI tools, which averaged just 1.8% (Mann–Whitney *U*, *p* = 0.003). This disparity is visually distinct in Figure 3, which illustrates that the vast majority of Generative and Ambient AI tools (Median: 0.0%) are currently operating as complete “black boxes” with no public technical disclosure.

To identify the specific drivers of this disparity, the reporting rates of individual metrics were compared between the Imaging and Generative sectors using Fisher's Exact Test (Table 2). The analysis revealed a distinct “Validation Gap,” where Imaging vendors were significantly more likely to provide evidence of scientific rigor. Regarding fundamental validity, 36.8% of Imaging vendors cited peer-reviewed evidence and reported specific accuracy metrics (e.g., sensitivity/specificity), compared to only 2.1% of Generative and Ambient AI vendors (*p* < 0.001 for both). While marketing claims of “high accuracy” were common across the industry, the reporting of statistically valid metrics was significantly concentrated in the imaging domain. Methodological transparency followed a similar pattern; the use of a defined, independent test set—distinct from the training data—was disclosed by 26.3% of Imaging vendors but only 2.1% of Generative and Ambient AI vendors (*p* = 0.006). Crucially for clinical decision-making, 15.8% of Imaging vendors reported confidence intervals to quantify uncertainty, whereas 0% of Generative and Ambient vendors did so (*p* = 0.021).

**Table 2:** Comparison of transparency reporting rates by clinical domain (Fisher's exact test).

**Transparency metric**	**Diagnostic imaging (*n* = 19)**	**Generative and ambient (*n* = 47)**	***p*-value**
**Validation and performance (the validation gap)**			
Peer-reviewed/accuracy metrics	36.8%	2.1%	**< 0.001**
Independent test set defined	26.3%	2.1%	**0.006**
Confidence intervals reported	15.8%	0.0%	**0.021**
**Safety, risk and demographics (systemic failure)**			
Risk mitigation/guardrails	5.3%	0.0%	1.0
Known failure modes	5.3%	0.0%	0.50
Bias/fairness evaluation	5.3%	0.0%	0.29
Signalment (demographics)	0.0%	2.1%	1.0

A significant “Validation Gap” exists between imaging and generative tools, while safety reporting remains universally low. Bold *p*-values indicate statistical significance (*p* < 0.05). Percentages reflect the proportion of vendors in that sector disclosing the specific metric in public documentation.

Despite the relatively higher reporting frequencies of the Imaging sector in validation metrics, both sectors demonstrated a systemic failure to report critical safety and demographic data. No significant difference was found between sectors regarding Risk Controls (*p* = 1.0), Bias/Fairness Evaluation (*p* = 0.29), or Known Failure Modes (*p* = 0.50), as reporting rates for these metrics were universally negligible (< 6% across both groups; Table 2).

Most notably, data transparency regarding patient applicability was virtually non-existent. Only one vendor (1.4%) disclosed the Signalment Distribution of their training data and provided Subgroup Performance analysis. This finding indicates that while some vendors provided evidence of model validation, nearly all failed to disclose the specific population the model was trained on, rendering it impossible for a clinician to assess whether the tool is appropriate for the specific patient phenotype under their care.

## 4 Discussion

### 4.1 The state of opacity in veterinary AI

To our knowledge, this study represents the first systematic audit of transparency in the commercial veterinary AI market. The results are consistent with the “regulatory vacuum” described in the Introduction and suggest that it has coincided with a market environment characterized by systemic opacity. With a mean transparency score of 6.4% and over 60% of vendors failing (Figure 2) to disclose a single validity or safety metric, the current landscape places veterinary professionals in a precarious position. Although the regulatory burden of validation nominally rests with the licensed practitioner, this audit indicates that the information required to meaningfully perform such validation is frequently unavailable in public-facing vendor documentation.

This creates a fundamental paradox: veterinarians are ethically and legally responsible for the tools they use, yet they are forced to adopt these tools largely on faith rather than evidence. Further, this data should not be obscured by paywalls or restrictive legal agreements; to enable informed purchasing decisions, veterinary professionals and clinic administrators require unrestricted access to validation metrics prior to entering the software procurement cycle.

This lack of transparency is particularly alarming because it has allowed the veterinary market to deviate dangerously from established clinical safety frameworks. Current models for AI scribe integration in human primary care describe a “staged maturation” process, where technology must prove reliable at automating documentation (Stage 1) and administrative workflows (Stage 2) before attempting reactive (Stage 3) or proactive (Stage 4) clinical decision support (43). In this model, “Stage 4” represents a future state where the AI proactively prompts clinicians regarding missed diagnoses or screening opportunities—a capability viewed as “achievable in the near future” but requiring rigorous validation to ensure safety (43).

Our results suggest that the veterinary AI market has inverted this safety progression. While human healthcare proceeds cautiously with Stage 1 “scribe” tools, recent evaluations demonstrate that this foundational technology remains prone to significant errors. Ha et al. found that no AI scribe in human primary care was consistently error-free, with frequent omissions of social history and critical details (29). Similarly, Biro et al. reported a 70% error rate in draft notes, noting that omission errors are particularly dangerous because they require the clinician to recall absent details from memory (30). Ambient AI scribes introduce a distinct cognitive safety risk: errors of omission are harder to detect than errors of commission, because missing details provide no salient cue and require effortful reconstruction of the encounter to verify. As a result, Human-in-the-Loop review is likely less effective than often assumed, consistent with evidence on automation bias, verification burden and emerging evaluations of ambient scribe failure modes (19, 31, 32).

Despite these documented failures at Stage 1, the unregulated nature of the veterinary market has allowed vendors to aggressively market Stage 4 capabilities. Veterinary tools frequently offer automated differential diagnosis generation, triage urgency scoring, and treatment plan recommendations. This creates a second, more dangerous paradox: the veterinary sector is deploying “Proactive Consultant” (Stage 4) features on top of “Passive Scribe” (Stage 1) technology that human medicine has deemed insufficiently reliable for unmonitored use. By “leapfrogging” the validation phase required for documentation, veterinary vendors are embedding unquantified risks into complex care delivery systems.

### 4.2 The transparency gap

Our results define the “Transparency Gap” as the industry-wide chasm between marketed capability and accessible evidence; veterinarians are sold tools with “Stage 4” proactive capabilities while being denied the basic data required to trust them. Secondarily, a distinct “Validation Gap” has emerged between sectors. While diagnostic imaging (a discriminative task) shows signs of maturing transparency, the burgeoning generative AI sector remains characterized by almost total opacity.

A critical finding of this study is the significant stratification of transparency by domain. The “Validation Gap” observed between Diagnostic Imaging and Generative and Ambient AI tools suggests a maturing market in radiology contrasted by a speculative, hype-driven expansion in generative AI. Diagnostic Imaging AI, having existed longer and drawing from established metrics in human radiomics (e.g., sensitivity, specificity, AUC), showed moderate adherence to validation standards. Nearly 37% of imaging vendors provided peer-reviewed evidence or performance metrics.

However, even where evidence exists, a risk of “Partial Validation” obscures the true capability of the product. Vendors in the imaging sector frequently cite peer-reviewed evidence for a specific, narrow task—such as the detection of pleural effusion or cardiomegaly—while marketing a comprehensive “AI Radiology” platform that claims to identify dozens of other pathologies. The existence of a high-quality study for one feature does not imply validation for the entire product suite. This “halo effect” can mislead clinicians into assuming that the rigorous validation of a single algorithm extends to the unvalidated remainder of the tool's capabilities.

In contrast, the rapidly expanding sector of Generative AI (LLMs and Scribes) operates with near-total opacity. We acknowledge that this disparity is partially driven by inherent technical differences between modalities. Validating a diagnostic imaging model is a relatively straightforward “discriminative” task with established ground truths (e.g., biopsy or expert consensus) and standard metrics. In contrast, validating Generative AI involves evaluating open-ended, non-deterministic outputs—a “generative” task where “correctness” is often subjective and harder to quantify. However, while this complexity explains a lag in performance reporting, it does not excuse the universal failure to disclose basic provenance data, such as training demographics, which requires no complex statistical machinery to report.

This failure is particularly concerning given the well-documented propensity of Large Language Models (LLMs) to “hallucinate” or confabulate medical information (33–38). The widespread integration of unevaluated LLMs into clinical workflows introduces a silent risk of error that may propagate into the permanent medical record without the safeguards of traditional software validation. Perhaps the most concerning finding of this audit is the universal failure to report training data demographics. Across all 71 vendors, only one product disclosed the signalment distribution (breed, age, sex) of its training or testing datasets. In veterinary medicine, biological variation is substantial; a diagnostic algorithm trained predominantly on adult Golden Retrievers may fail to generalize to a geriatric Chihuahua or a distinct feline phenotype. Without knowledge of the “intended use population” (the specific cohorts the model was trained on), clinicians cannot assess whether the AI is appropriate for the patient currently on the table. This lack of demographic transparency makes it impossible to detect potential algorithmic bias, risking a scenario where AI tools systematically underperform on underrepresented breeds or species, leading to disparities in care that remain invisible to the user.

The absence of safety reporting—specifically regarding failure modes and risk mitigation—suggests an industry-wide reliance on the “Human-in-the-Loop” defense. Vendors often suggest that strict validation is unnecessary because the veterinarian is the ultimate decision-maker. However, this argument fails to account for the crucial distinction between a “Human-in-the-Loop” and a “Domain-Expert-in-the-Loop.” Research in human medicine suggests that while domain experts (e.g., radiologists) may retain a “protective effect” against automation bias when using AI in their field, this protection is lost when the user is a generalist relying on a specialist tool. For a general practitioner using an AI radiologist or an automated cardiologist, the “safety net” of domain expertise is absent (39–41).

Currently, vendors undermine the veterinarian's ability to act as a safeguard by ignoring the cognitive impact of automation bias, where clinicians are statistically likely to defer to an automated system's judgment, especially when that system provides high-confidence outputs without uncertainty intervals (19, 38). Our audit found that 0% of Generative and Ambient vendors and only 15.8% of Imaging vendors provided confidence intervals or other metrics regarding the statistical uncertainty of their model. By presenting AI outputs as definitive facts rather than probabilistic estimates, and by failing to disclose known failure modes (when the model is likely to be wrong), vendors compromise clinical safety. Transparency regarding model uncertainty should be viewed not as an admission of limitation, but as a critical mechanism for building clinical trust and enabling safe human-in-the-loop decision-making.

### 4.3 Limitations

This study was limited to publicly available documentation. It is possible that vendors maintain robust internal validation data that is shared only under non-disclosure agreements or during the sales process. However, the premise of “Good Machine Learning Practice” is that transparency builds trust; hiding evidence behind paywalls restricts independent academic scrutiny and broadly inhibits the profession's ability to establish standards of care.

A notable methodological limitation is the use of a single rater for the primary audit. While intra-rater reliability was rigorously established via a blinded re-audit of 20% of the sample (κ>0.90), the absence of a second independent reviewer for the full dataset limits the assessment of inter-rater reliability. Although the “Low-Inference” scoring protocol was designed to minimize subjectivity by requiring binary, verifiable evidence for all metrics, future studies would benefit from a multi-rater design to further validate these findings.

Additionally, the AI market is highly volatile, products and documentation change rapidly. This audit represents a cross-sectional snapshot of the market as of November 2025. While the use of a narrow 7-day search window controlled for internal variability during the study, it also highlights a limitation of the field: the veterinary AI market is highly volatile. Documentation is dynamic, and products audited as “opaque” during this window may have subsequently published validation data or altered their claims post-audit.

Finally, the VATI instrument, while mapped to established human healthcare frameworks, has not undergone formal content validation by an independent expert panel; future studies should prioritize this step before broader application.

### 4.4 Ethical implications and professional liability

The sale and clinical deployment of unvalidated SaMD raises profound ethical concerns. In human healthcare, the use of an unproven medical device on a patient would typically constitute clinical research, requiring Institutional Review Board (IRB) oversight and informed consent. In the current veterinary market, however, unvalidated AI tools are routinely deployed on client-owned animals outside of any research framework. This practice effectively offloads the risk of product testing onto the patient and the liability onto the practitioner (10, 21).

Furthermore, the opaque nature of these tools creates a conflict with the principles of the Veterinarian-Client-Patient Relationship (VCPR). When a veterinarian utilizes a specialist consultant (e.g., a board-certified radiologist), the consultant shares professional accountability for their advice. In contrast, many AI tools effectively function as “consultants”—offering differential diagnoses, treatment plans, and triage scores—yet lack both legal accountability and transparency. If a clinician cannot interrogate the “consultant's” reasoning because it is a “black box,” they cannot fulfill their professional obligation to verify the medical appropriateness of the recommendation. Consequently, the veterinarian assumes full liability for an algorithmic decision they did not make and cannot explain, fundamentally undermining the informed consent provided to the client (3, 10).

### 4.5 Conclusion and future directions

The commercial veterinary AI market currently operates as a “black box,” characterized by a level of opacity that would be unacceptable in human healthcare. To bridge the gap between technological potential and clinical safety, the profession must move beyond passive consumption of these tools and demand active accountability. First, we propose the development and pilot testing of a standardized “Model Card” framework adapted for veterinary medicine. Whether derived from the Coalition for Health AI (CHAI) standards or developed as a novel veterinary-specific standard, this framework must function as a “nutrition label” for AI (42). It should require vendors to disclose the “ingredients” (training data demographics), “nutritional value” (performance metrics on external test sets), and “allergens” (known limitations and failure modes). Second, self-regulation is insufficient. We propose the establishment of independent third-party accreditation—a “VetAI-Trust”—analogous to HITRUST in health IT security. Such a body would verify vendor claims and grant accreditation only to tools that demonstrate reproducible safety and efficacy, shifting the burden of validation from the busy practitioner to the vendor. Third, transparency is effective only if the end-user can interpret it. Veterinary curricula and continuing education must evolve to prioritize AI literacy, equipping clinicians with the skills to critically evaluate model limitations and interpret probabilistic outputs within a clinical context. Finally, future development must move beyond generic architectures toward signalment-conditioned models. Current “one-size-fits-all” approaches often fail to account for the immense physiological diversity between species and breeds. The next generation of veterinary AI must explicitly incorporate diverse signalment data—conditioning outputs on species, breed, age, and sex—to ensure that predictive performance is robust across the heterogeneity of the veterinary patient population. Only through standardized transparency and signalment-aware design can we transition from a market of hype to a discipline of evidence-based computational medicine.

## Data Availability

The raw data supporting the conclusions of this article will be made available by the authors, without undue reservation.

## References

[1] Akinsulie OC Idris I Aliyu VA Shahzad S Banwo OG Ogunleye SC . The potential application of artificial intelligence in veterinary clinical practice and biomedical research. Front Vet Sci. (2024) 11:1347550. doi: 10.3389/fvets.2024.1347550 38356661 PMC10864457

[2] Appleby RB Difazio M Cassel N Hennessey R Basran PS. American College of Veterinary Radiology and European College of Veterinary Diagnostic Imaging position statement on artificial intelligence. J Am Vet Med Assoc. (2025) 263:773–6. doi: 10.2460/javma.25.01.0027 40107235

[3] American Association of Veterinary State Boards. Regulatory Considerations of the USE of Artificial Intelligence in Veterinary Medicine. (2025). Available online at: https://www.aavsb.org/wp-content/uploads/2025/08/AAVSB-AI-Guidance-Whitepaper.pdf (Accessed November 26, 2025).

[4] Albergante L O'Flynn C De Meyer G. Artificial intelligence is beginning to create value for selected small animal veterinary applications while remaining immature for others. J Am Vet Med Assoc. (2025) 263:388–94. doi: 10.2460/javma.24.09.0617 39746305

[5] Bouchemla F Akchurin SV Akchurina IV Dyulger GP Latynina ES Grecheneva AV. Artificial intelligence feasibility in veterinary medicine: a systematic review. Vet World. (2023) 16:2143–9. doi: 10.14202/vetworld.2023.2143-2149 38023280 PMC10668547

[6] Basran PS Appleby RB. The unmet potential of artificial intelligence in veterinary medicine. Am J Vet Res. (2022) 83:385–92. doi: 10.2460/ajvr.22.03.0038 35353711

[7] Joslyn S Alexander K. Evaluating artificial intelligence algorithms for use in veterinary radiology. Vet Radiol Ultrasound. (2022) 63(Suppl. 1):871–9. doi: 10.1111/vru.13159 36514228

[8] Lustgarten JL Zehnder A Shipman W Gancher E Webb TL. Veterinary informatics: forging the future between veterinary medicine, human medicine, and One Health initiatives a joint paper by the Association for Veterinary Informatics (AVI) and the CTSA One Health Alliance (COHA). JAMIA Open. (2020) 3:306–17. doi: 10.1093/jamiaopen/ooaa005 32734172 PMC7382640

[9] Bellamy JEC. Artificial intelligence in veterinary medicine requires regulation. Can Vet J. 64:968–70. 37780472 PMC10506349

[10] Cohen EB Gordon IK. First, do no harm. Ethical and legal issues of artificial intelligence and machine learning in veterinary radiology and radiation oncology. Vet Radiol Ultrasound. (2022) 63(Suppl. 1):840–50. doi: 10.1111/vru.13171 36514231 PMC10107688

[11] Guiding Principles – GMLP. Available online at: https://www.fda.gov/media/153486/download (Accessed November 26, 2025).

[12] Center for Veterinary Medicine. How FDA Regulates Animal Devices. Silver Spring, MD: FDA (2025). (Accessed November 26, 2025).

[13] Center for Devices and Radiological Health. Software as a Medical Device (SaMD). Silver Spring, MD: FDA (2025). Available online at: https://www.fda.gov/medical-devices/software-medical-device-samd/artificial-intelligence-enabled-medical-devices (Accessed November 26, 2025).

[14] Kiseleva A Kotzinos D De Hert P. Transparency of AI in healthcare as a multilayered system of accountabilities: between legal requirements and technical limitations. Front Artif Intell. (2022) 5:879603. doi: 10.3389/frai.2022.879603 35707765 PMC9189302

[15] Nagendran M Chen Y Lovejoy CA Gordon AC Komorowski M Harvey H . Artificial intelligence versus clinicians: systematic review of design, reporting standards, and claims of deep learning studies. BMJ. (2020) 368:m689. doi: 10.1136/bmj.m689 32213531 PMC7190037

[16] CVMA. CVMA Policy on the Use of Artificial Intelligence in Veterinary Medicine. Sacramento, CA: CVMA (2024). Available online at: https://cvma.net/about-us/policies/cvma-policy-on-the-use-of-artificial-intelligence-in-veterinary-medicine/ (Accessed November 26, 2025).

[17] Xu H Shuttleworth KMJ. Medical artificial intelligence and the black box problem: a view based on the ethical principle of “do no harm”. Intell Med. (2024) 4:52–7. doi: 10.1016/j.imed.2023.08.001

[18] Amann J Blasimme A Vayena E Frey D Madai VI Precise4Q Consortium. Explainability for artificial intelligence in healthcare: a multidisciplinary perspective. BMC Med Inform Decis Mak. (2020) 20:310. doi: 10.1186/s12911-020-01332-6 33256715 PMC7706019

[19] Goddard K Roudsari A Wyatt JC. Automation bias: a systematic review of frequency, effect mediators, and mitigators. J Am Med Inform Assoc. (2012) 19:121–7. doi: 10.1136/amiajnl-2011-000089 21685142 PMC3240751

[20] Joslyn SK Faulkner J Ma D Appleby R. Commentary: comparison of radiological interpretation made by veterinary radiologists and state-of-the-art commercial AI software for canine and feline radiographic studies. Front Vet Sci. (2025) 12:1615947. doi: 10.3389/fvets.2025.1615947 40636808 PMC12238720

[21] Basran PS Appleby RB. What's in the box? A toolbox for safe deployment of artificial intelligence in veterinary medicine. J Am Vet Med Assoc. (2024) 262:1090–8. doi: 10.2460/javma.24.01.0027 38599232

[22] Burti S Zotti A Banzato T. Role of AI in diagnostic imaging error reduction. Front Vet Sci. (2024) 11:1437284. doi: 10.3389/fvets.2024.1437284 39280838 PMC11392848

[23] Page MJ McKenzie JE Bossuyt PM Boutron I Hoffmann TC Mulrow CD . The PRISMA 2020 statement: an updated guideline for reporting systematic reviews. BMJ. (2021) 372:n71. doi: 10.1136/bmj.n71 33782057 PMC8005924

[24] Office of the National Coordinator for Health Information Technology. Health data, technology, and interoperability: certification program updates, algorithm transparency, and information sharing. Fed Regist. (2024) 89:1192–436.

[25] Liu X Rivera SC Moher D Calvert MJ Denniston AK SPIRIT-AI and CONSORT-AI Working Group. Reporting guidelines for clinical trial reports for interventions involving artificial intelligence: the CONSORT-AI extension. Lancet Digit Health. (2020) 2:e537–48. doi: 10.1136/bmj.m3164 33328048 PMC8183333

[26] Coalition for Health AI. Applied Model Card. Chai.org. (2025). Available online at: https://www.chai.org/workgroup/applied-model (Accessed December 4, 2025).

[27] International Medical Device Regulators Forum. Software as a Medical Device: Possible Framework for Risk Categorization and Corresponding Considerations. Singapore: International Medical Device Regulators Forum (2014).

[28] Center for Devices and Radiological Health. Global Approach to Software as a Medical Device. Silver Spring, MD: FDA (2022). Available online at: https://www.fda.gov/medical-devices/software-medical-device-samd/global-approach-software-medical-device (Accessed December 4, 2025).

[29] Ha E Choon-Kon-Yune I Murray L Luan S Montague E Bhattacharyya O . Evaluating the usability, technical performance, and accuracy of artificial intelligence scribes for primary care: competitive analysis. JMIR Human Factors. (2025) 12:e71434. doi: 10.2196/71434 40700466 PMC12309782

[30] Biro J Handley JL Cobb NK Kottamasu V Collins J Krevat S . Accuracy and safety of AI enabled scribe technology. J Med Internet Res. (2024) 27:e64993. doi: 10.2196/64993 PMC11811668 39869899

[31] Lyell D Coiera E. Automation bias and verification complexity: a systematic review. J Am Med Inform Assoc. (2017) 24:423–31. doi: 10.1093/jamia/ocw105 27516495 PMC7651899

[32] Topaz M Peltonen LM Zhang Z. Beyond human ears: navigating the uncharted risks of AI scribes in clinical practice. NPJ Digit Med. (2025) 8:569. doi: 10.1038/s41746-025-01895-6 40993221 PMC12460601

[33] Huang L Ma W Zhong W Feng Z Wang H Chen Q . A survey on hallucination in large language models: principles, taxonomy, challenges, and open questions. ACM Trans Inf Syst. (2025) 43:1–55. doi: 10.1145/3703155

[34] Kim Y Jeong H Park C Alhamoud K Grau C Jung M . Medical hallucination in foundation models and their impact on healthcare. medRxiv [Preprint]. (2025). doi: 10.1101/2025.02.28.25323115

[35] Goodman RS Patrinely JR Stone CA Jr Zimmerman E Donald RR Chang SS . Accuracy and reliability of chatbot responses to physician questions. JAMA Netw Open. (2023) 6:e2336483. doi: 10.1001/jamanetworkopen.2023.36483 37782499 PMC10546234

[36] Johnson D Goodman R Patrinely J Stone C Zimmerman E Donald R . Assessing the accuracy and reliability of AI-generated medical responses: an evaluation of the Chat-GPT model. Res Sq. (2023) rs.3.rs-2566942. doi: 10.21203/rs.3.rs-2566942/v1 36909565 PMC10002821

[37] Weinberg J Goldhardt J Patterson S. Kepros J. Assessment of accuracy of an early artificial intelligence large language model at summarizing medical literature: ChatGPT 35 vs ChatGPT 40. J Med Artif Intell. (2024) 7:33. doi: 10.21037/jmai-24-48

[38] Garg AX Adhikari NK McDonald H Rosas-Arellano MP Devereaux PJ Beyene J . Effects of computerized clinical decision support systems on practitioner performance and patient outcomes a systematic review. JAMA. (2005) 293:1223–38. doi: 10.1001/jama.293.10.1223 15755945

[39] Dratsch T Chen X Rezazade Mehrizi M Kloeckner R Mähringer-Kunz A Püsken M . Automation bias in mammography: the impact of artificial intelligence BI-RADS suggestions on reader performance. Radiology. (2023) 307:e222176. doi: 10.1148/radiol.222176 37129490

[40] Bond RR Novotny T Andrsova I Koc L Sisakova M Finlay D . Automation bias in medicine: the influence of automated diagnoses on interpreter accuracy and uncertainty when reading electrocardiograms. J Electrocardiol. (2018) 51:S6–11. doi: 10.1016/j.jelectrocard.2018.08.007 30122457

[41] Khera R Simon MA Ross JS. Automation bias and assistive AI: risk of harm from AI-driven clinical decision support. JAMA. (2023) 330:2255–7. doi: 10.1001/jama.2023.22557 38112824

[42] Mitchell M Wu S Zaldivar A Barnes P Vasserman L Hutchinson B . Model cards for model reporting. arXiv [Preprint]. (2019) arXiv:1810.03993. Available online at: https://arxiv.org/pdf/1810.03993 (Accessed November 26, 2025).

[43] Seth P Carretas R Rudzicz F. The utility and implications of ambient scribe in primary care. JMIR AI. (2024) 3:e57673. doi: 10.2196/57673 39365655 PMC11489790

