# Supplementary material for: A systematic audit of transparency and validation disclosure in commercial veterinary artificial intelligence
Source: Front Vet Sci. 2026 Mar 5;13:1761038. doi: 10.3389/fvets.2026.1761038 (PMC12999420; doi:10.3389/fvets.2026.1761038)
Supplement: Supplementary file 1 [file Data_Sheet_1.pdf]

Supplementary Material

Supplementary Appendix S1. Automated Web Archiving Pipeline

To obtain reproducible, time-stamped snapshots of veterinary AI–related websites, we implemented an automated web-archiving pipeline using the GNU `wget` utility (v1.21) orchestrated via Python. All sites were archived as they existed at the time of retrieval.

A list of 71 target URLs was compiled in a CSV file. For each URL, the pipeline initiated an independent `wget` process configured to (i) create a complete offline mirror of the website and (ii) generate a WARC (Web ARChive) file suitable for long-term preservation. The following flags were applied uniformly across all crawls:

- `--mirror` (recursive retrieval with timestamping)
- `--page-requisites` (capture all assets required for local rendering)
- `--convert-links` and `--adjust-extension` (produce navigable offline HTML)
- --warc-file=<path> (write a standards-compliant WARC file)

Each website was assigned a unique, filesystem-safe identifier. Archived site content was stored in a dedicated directory (sites/<site_id>/), while corresponding compressed WARC files were stored separately (warcs/<site_id>.warc.gz). All crawler output, including HTTP responses and errors, was logged to a per-site `wget.log` file to support auditing and verification.

To improve efficiency while maintaining polite crawling behavior, downloads were executed in parallel using Python's `concurrent.futures.ThreadPoolExecutor` with a worker pool of four concurrent processes. Each crawl was independent and shared no state. For every site, the pipeline recorded the original URL, crawl timestamp (UTC), output directory, WARC file path, and `wget` exit code in a manifest CSV file to ensure full reproducibility.

This procedure produced a versioned, research-grade archive of each website, enabling subsequent analysis without reliance on live online content and ensuring that all materials reflected their state at the time of capture.

Supplementary Appendix S2. The Veterinary AI Transparency Index (VATI) Adjudication Rubric

Scoring Protocol

Each metric is scored as binary: 0 (Non-Disclosure) or 1 (Disclosure). To minimize interpretative bias, a strict *low-inference* protocol is applied.

- **Score = 0 (Non-Disclosure):** Assigned if the information is missing or if the vendor provides only qualitative marketing assertions (e.g., "high accuracy," "state-of-the-art," "diverse dataset") without supporting evidence.

- **Score = 1 (Disclosure):** Assigned only if the vendor provides specific, quantifiable, or verifiable evidence (e.g., numerical values, dates, citations, or explicit policy statements).

Table 1: Veterinary AI Transparency Index (VATI) metrics and adjudication criteria.

#	VATI Metric	Definition	Criteria for Score = 0 (Non-Disclosure)	Criteria for Score = 1 (Disclosure)
1	Training Data Volume	Total size/count of the dataset used to develop the model.	Vague claims (e.g., "large dataset," "millions of data points").	Specific integers (e.g., "15,000 images," "50,000 clinical notes").
2	Data Provenance (Source)	Origin of the training data.	"Proprietary data," "partner clinics," "global network."	Named sources (e.g., university, hospital, country/region).
3	Patient Demographics	Biological characteristics of the training population (signalment).	"Diverse breeds," "all sizes."	Distribution reported for at least one of breed, age, sex, or species.
4	Geographic/Site Diversity	Distinct collection sites or geographic variance.	"Global data," "multi-center."	Specific site count or list of countries/states.
5	Data Temporality	Time period during which training data were collected.	"Recent data," "modern data."	Explicit year range or collection date.
6	Reference Standard	Ground truth used to label the training data.	"Expert review," "labeled by vets."	Specific standard (e.g., board-certified consensus, histopathology, PCR).
7	Data Exclusion Criteria	Criteria for removing poor-quality data.	Silent or generic "quality controlled."	Explicit rules (e.g., motion blur excluded, incomplete records removed).
8	Performance Metrics	Quantitative measures of model accuracy.	"High accuracy," marketing-only claims.	Specific metrics on a test set (e.g., sensitivity, specificity, AUC, F1).
9	Confidence Intervals	Statistical uncertainty around performance metrics.	Point estimates only.	Confidence intervals or standard deviations reported.
10	Independence of Test Set	Confirmation that test data were distinct from training data.	Silent or implied split.	Explicit statement of held-out or unseen test set.
11	External Validity	Testing on data from a new source or site.	Internal testing only.	Evaluation on external clinic, device, or time period.
12	Subgroup Analysis	Performance by patient subgroup.	Overall accuracy only.	Stratified performance (e.g., by breed, species, lesion size).

#	VATI Metric	Definition	Criteria for Score = 0 (Non-Disclosure)	Criteria for Score = 1 (Disclosure)
13	Human Benchmark	Comparison with veterinary professionals.	Qualitative claims (e.g., "better than vets").	Quantitative head-to-head comparison with clinicians.
14	Peer-Reviewed Validation	Evidence of external scientific scrutiny.	White papers, blogs, internal reports.	Citation or DOI to peer-reviewed publication.
15	Limitations / Failures	Disclosure of known failure modes.	"Use with caution."	Explicit contraindications or failure cases.
16	Bias Evaluation	Assessment of performance disparities.	Generic "fair" or "unbiased" claims.	Explicit testing for group-level performance differences.
17	OOD Handling	Handling of out-of-distribution inputs.	Silent.	Mechanism to reject or flag OOD inputs.
18	Risk Mitigation	Guardrails to prevent unsafe outputs.	Generic "human in the loop."	Specific safety mechanisms or constraints.
19	Post-Market Feedback	Mechanism for users to report errors.	Generic contact form or email.	Dedicated clinical error or adverse-event reporting pathway.
20	Model Design	Technical architecture of the AI system.	"AI," "deep learning," "proprietary algorithm."	Specific architecture named (e.g., ResNet-50, Transformer, U-Net).
21	Explainability	Features aiding user interpretation.	Black-box output only.	Visual or textual explanations (e.g., heatmaps, bounding boxes).
22	Output Confidence	Probabilistic confidence provided to the user.	Binary output or class label only.	Probability score, confidence bar, or risk score displayed.
23	Model Card	Standardized technical documentation.	Absent.	Explicitly labeled model card or data sheet available.
24	Regulatory Clearance	Status with regulatory bodies.	Silent or "compliant."	Explicit clearance or regulatory status stated.
25	User Instructions	Guidance on input requirements.	"Easy to use."	Detailed input specifications provided.

Supplementary Appendix S3. Included Commercial AI Vendors

Table 2: List of all 71 commercially available veterinary AI tools included in the audit, with their identification source and public website.

Vendor Name	Source	Website	Type
Generative and Ambient AI			
AI For Pet	VMX	`https://www.aiforpet.com`	Generative / Ambient
CompanAIn	LinkedIn	`https://www.companain.ai/`	Generative / Ambient
CoVet Veterinary CoPilot	Apple	`https://www.co.vet/`	Generative / Ambient
Chart Hound	CrunchBase	`https://www.charthound.ai/`	Generative / Ambient
DaySmart Vet Daisy	VMX	`https://www.daysmart.com/vet/daisy-ai/`	Generative / Ambient
Digitail	VMX	`https://www.digitail.com/`	Generative / Ambient
Dragon Veterinary	VMX	`https://www.dragonveterinary.com/vetbuddy-home`	Generative / Ambient
FydoDx	VMX	`https://www.fydodx.com/`	Generative / Ambient
Happy Doc	CrunchBase	`https://www.happydoc.ai/`	Generative / Ambient
Heidi	Google	`https://www.heidihealth.com/en-us/solutions/veterinarians`	Generative / Ambient
Laika	Google	`https://www.laika.aitemsolutions.com/home`	Generative / Ambient
Lupa Pets	CrunchBase	`https://www.lupapets.com`	Generative / Ambient
NectarVet	VMX	`https://www.nectarvet.com`	Generative / Ambient
NXVET	AVMA	`https://www.nx.vet`	Generative / Ambient
Otto	VMX	`https://www.otto.vet/`	Generative / Ambient
PawfectNotes	VMX	`https://www.pawfectnotes.com`	Generative / Ambient
PawSmart	CrunchBase	`https://www.pawsmart.ai/`	Generative / Ambient
PetDesk	VMX	`https://www.petdesk.com/veterinary-ai-transcription-platform/`	Generative / Ambient
PetsApp	VMX	`https://www.petsapp.com/`	Generative / Ambient
PetWise	VMX	`https://www.petwise.vet`	Generative / Ambient
Pivio	CrunchBase	`https://www.pivio.ai`	Generative / Ambient
Provet Cloud	VMX	`https://www.provet.com/product/clinical-ai`	Generative / Ambient
PupPilot	VMX	`https://www.puppilot.co/our-story`	Generative / Ambient
ScribbleVet	Apple	`https://www.scribblevet.com/`	Generative / Ambient
Scribenote	Apple	`https://www.scribenote.com/`	Generative / Ambient
Scribevet	Apple	`https://www.scribvet.com/`	Generative / Ambient
Scritch	CrunchBase	`https://www.scritchai.com`	Generative / Ambient
Shepherd Vet. Solutions	VMX	`https://www.shepherd.vet/`	Generative / Ambient
Soapnote.vet	Apple	`https://www.soapnote.vet/`	Generative / Ambient
Sofie AI	Google	`http://www.pattersonvet.com/software/medical-assistance/sofie-veterinary-medical-search-tool`	Generative / Ambient
Talkatoo	Apple	`https://www.talkatoo.com/`	Generative / Ambient
Talkingvet	VMX	`https://www.talkingvet.com/`	Generative / Ambient
VEA	CrunchBase	`https://www.veaforvets.com`	Generative / Ambient
Vetbrain	LinkedIn	`https://www.vetbrain.ro`	Generative / Ambient
VetGeni	VMX	`https://www.vetgeni.com`	Generative / Ambient

Continued on next page

Continued from previous page

Vendor Name	Source	Website	Type
VetHubb	CrunchBase	`https://www.vethubb.ai/`	Generative / Ambient
Vetnio	CrunchBase	`https://vetnio.com/`	Generative / Ambient
Vetnotes	Google	`https://www.vetnotes.com`	Generative / Ambient
Vetpin AI	Google	`https://www.medipin.ai/en/`	Generative / Ambient
VetPulse	AVMA	`https://www.myvetpulse.com/`	Generative / Ambient
VetRec	Apple	`https://www.vetrec.io/`	Generative / Ambient
VetScribe	CrunchBase	`https://www.vetscribe.co`	Generative / Ambient
VetSkribe	LinkedIn	`https://www.vetskribe.com/`	Generative / Ambient
Vetspire	CrunchBase	`https://www.vetspire.com`	Generative / Ambient
VetVise	CrunchBase	`https://www.vetvise.com`	Generative / Ambient
ViggoVet	CrunchBase	`https://www.viggo.vet`	Generative / Ambient
Vital Vet AI	LinkedIn	`https://www.vitalvetai.ca`	Generative / Ambient
Imaging			
Aiforia	Google	`https://www.aiforia.com/veterinary-diagnostics`	Imaging
Alphaion	VMX	`https://www.alphaionvet.com/pages/alphascan`	Imaging
Antech RapidRead	Google	`https://www.antechdiagnostics.com/imaging-services/rapidread/`	Imaging
Clarius Mobile Health	VMX	`https://www.clarius.com/`	Imaging
DiagnoVET	CrunchBase	`https://www.diagnovet.ai`	Imaging
Idexx	ACVR	`https://www.idexx.com/en/`	Imaging
MetronMind	CrunchBase	`https://www.metronmind.com`	Imaging
Micron AgriTech	CrunchBase	`https://www.micronagritech.com/`	Imaging
Moichor	CrunchBase	`https://www.moichor.com`	Imaging
MyVet Imaging	VMX	`https://www.myvetimaging.com/`	Imaging
Parasight System Inc	VMX	`http://www.parasightsystem.com/our-products`	Imaging
Picoxia	Google	`https://www.picoxia.com/en/`	Imaging
Radimal	VMX	`https://www.radimal.ai`	Imaging
SignalPET	CrunchBase	`https://www.signalpet.com/`	Imaging
VETEL DIAGNOSTICS	Google	`https://www.veteldiagnostics.com/`	Imaging
VetEye	Google	`https://www.veteyeapp.com/#feature`	Imaging
Vetology.net	CrunchBase	`https://www.vetology.net/`	Imaging
Xcaliber	Google	`https://www.xcaliber.ai/`	Imaging
Zoetis	VMX	`https://www.zoetis.com/products-and-science/diagnostics`	Imaging
Specialized / Other			
FidoCure	VMX	`https://www.fidocure.com/`	Specialized / Other
HT Vista	CrunchBase	`https://www.ht-vet.com/technology/`	Specialized / Other
ImpriMed	CrunchBase	`https://www.imprimedicine.com/personalized-prediction-profile`	Specialized / Other
Pointer Health	AVMA	`https://www.pointerhealth.com/`	Specialized / Other
Rokit Healthcare	AVMA	`https://www.rokithealthcare.com/kr`	Specialized / Other
